# Ginsenoside Rk1 Induces Apoptosis in Neuroblastoma Cells Through Loss of Mitochondrial Membrane Potential and Activation of Caspases

**DOI:** 10.3390/ijms20051213

**Published:** 2019-03-11

**Authors:** Jung-Mi Oh, Jeongwoo Lee, Wan-Taek Im, Sungkun Chun

**Affiliations:** 1 Department of Physiology, Chonbuk National University Medical School, Jeonju 54907, Korea; biojmi@jbnu.ac.kr; 2 Department of Anesthesiology and Pain Medicine, Chonbuk National University Hospital, Jeonju 54907, Korea; jw88lee@gmail.com; 3 Department of Biotechnology, Hankyoung National University, Anseong 17579, Korea; wandra@hknu.ac.kr; 4 Research Institute of Clinical Medicine of Chonbuk National University, Jeonju 54907, Korea; 5 Biomedical Research Institute of Chonbuk National University Hospital, Jeonju 54907, Korea

**Keywords:** apoptosis, ginsenoside Rk1, metastasis, neuroblastoma, proliferation

## Abstract

Neuroblastoma (NB) is the most common childhood cancer, with a very poor prognosis. More than 60% of children with NB die within five years; therefore, a more effective therapy for NB is required. Although ginsenoside has been shown to significantly inhibit the growth of various cancers, the effect of ginsenoside Rk1 on neuroblastoma has not been known yet. Hence, we examined the anticancer effects of highly pure Rk1 on neuroblastoma cell lines. The apoptotic effects of Rk1 on neuroblastoma cells were examined using cell viability assay, flow cytometry and cell staining assay, and the change in gene expression levels were analysed using RT-PCR, western blots, and immunohistochemistry. The metastatic effect of Rk1 was monitored by wound healing assay, invasion and migration with Matrigels. Rk1 inhibited neuroblastoma cell viability dose-dependently. Rk1-induced apoptosis was investigated through nuclear condensation and mitochondrial membrane potential loss, and it showed that Rk1 can induce cell cycle arrest at the G0/G1 phase but also inhibit the metastatic ability of neuroblastoma cells. Moreover, Rk1 (30 mg/kg) injections markedly inhibited xenograft tumor growth. These findings demonstrate that Rk1 might be valuable in the development of anti-cancer agents for neuroblastoma treatment.

## 1. Introduction

Neuroblastoma is the most common pediatric surgical malignancy, accounting for 6-8% of all pediatric tumors and more than 15% of pediatric cancer deaths. More than 50% of these tumors occur in children younger than two years [1]. Neuroblastoma can be found anywhere in the sympathetic neural tissue; because of its heterogeneity at diagnosis, neuroblastoma is generally progressive and highly malignant. Neuroblastoma also has the potential to metastasize to other organs; approximately 50% of patients are diagnosed with high-risk disease. High-dose chemotherapy using autologous stem cell transplantation significantly improves the symptoms of metastatic neuroblastoma, but it has many side-effects [2].

Despite intensive multimodal therapy (chemotherapy and surgery), neuroblastoma still has a poor prognosis for children with advanced or metastatic disease with a long-term survival rate of less than 40%. Tumorigenesis and malignant transformation are caused by overexpression of the cell survival pathway and normal cellular senescence or apoptosis. Thus, manipulation of the cell survival pathway can reduce the malignant potential of these tumors and provide a pathway to develop new therapies [3]. 

Apoptosis is a selective physiological process regulating the ratio of cell proliferation and cell death. For that reason, when apoptosis has not happened exactly as it should, it can be contributable to tumorigenesis. Therefore, targeting of key modulators on apoptosis could be a good strategy for developing cancer therapies [4].

*Panax ginseng Meyer* (ginseng) is a well-known natural product that has been used to treat diseases since ancient times. Among ginseng products, ginsenosides are regarded as the major active compound, and studies over the last decade have shown that they have anti-inflammation, neuroprotection, anti-metastasis, and anti-cancer effects [5,6,7,8].

The characteristics of ginsenosides that affect apoptosis in cancer cells have been studied because they have strong cytotoxicity, but low polarity. Several reports have demonstrated the anti-cancer properties of ginsenosides, including inhibition of tumor angiogenesis and metastasis, but also induction of apoptosis in several typical cancer types, such as lung [8], breast [9,10], colorectal cancer cells [11,12], as well as neuroblastoma cells [13,14].

Among those ginsenosides, the Rk1 compound is shown as rare saponin isolated from Sun Ginseng (SG). SG undergoes a novel type of processing that significantly strengthens the unique active ingredients in red ginseng. This enhanced anti-tumor activity results from the generation of ginsenosides by a heating process with SG [15,16]. These rare ginsenosides (minor ginsenosides) are commonly used for ginseng medicine and health foods. Nonetheless, the amount of these minor ginsenosides is small, because it is difficult to be extracted [17]. 

Rk1 was recently shown to have an anti-tumor effect in studies on human hepatocellular carcinoma cells [18] and human melanoma cells [19]. Although Rk1 has cytotoxic activity in some cancer cells in addition to an apoptotic effect, its mechanism of action is still unknown in neuroblastoma cells. Therefore, we isolated ginsenoside Rk1 from red ginseng and investigated its anti-cancer effects in the neuroblastoma cell lines in this study. We also examined these effects of Rk1 in vivo in nude mice. In conclusion, our findings suggest that Rk1 exerts anti-cancer effects through the induction of apoptosis and suppression of cell proliferation in neuroblastoma cell lines. 

## 2. Results

### 2.1. Rk1 Induces Reduction of Viability in Neuroblastoma Cells

**Figure 1:**
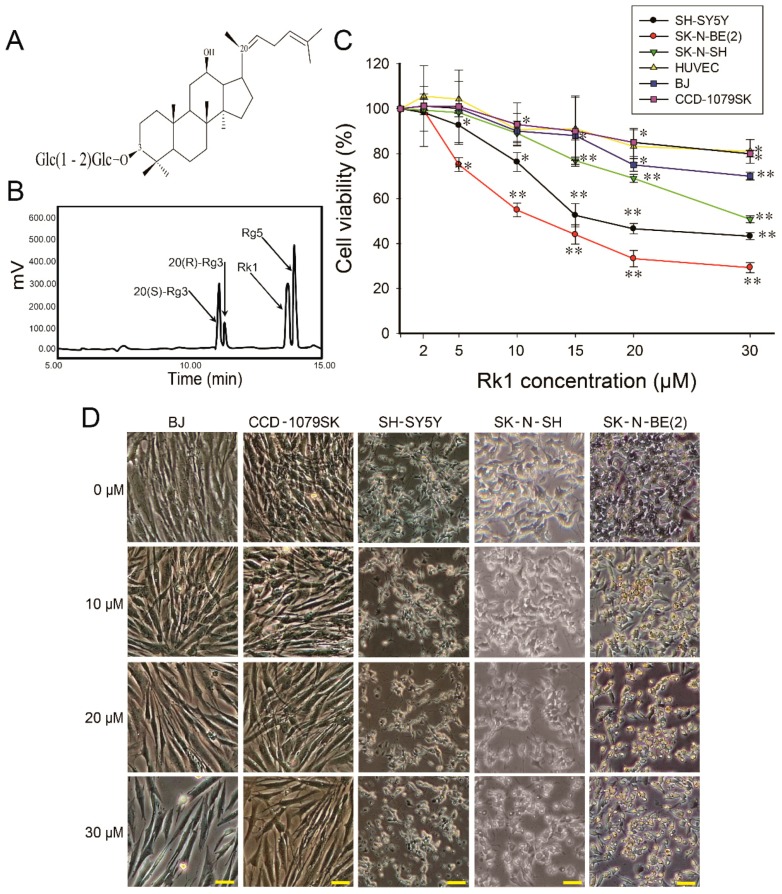
Growth inhibitory effect of Rk1 on neuroblastoma cells. (**A**) Chemical structure of Rk1. (**B**) HPLC analysis of the transformation for Rk1. The chromatographic graphic peaks were identified by comparison with the reference compounds. (**C**) Cell viability was determined by MTT assay. Data are presented as the mean ± SD of three independent experiments. *p* < 0.05 (*) or *p* < 0.01 (**) versus control (Rk1-untreated). (**D**) Morphologic change of cells was observed by microscopy. Scale bar: 50 μm.

To investigate the anticancer effect on neuroblastoma cell lines, we purified highly pure Rk1 from Korean ginseng (Figure 1B); Figure 1A shows the structure of Rk1. To investigate whether Rk1 exerts a cytotoxic effect, three neuroblastoma cell lines [SK-N-BE(2) (S-type), SK-N-SH (mixture of N and S-type), and SH-SY5Y (N-type) cells] and three normal cell lines (BJ, CCD-1079SK, and HUVEC) were treated at various concentrations of Rk1 (0, 2, 5, 10, 15, 20 and 30 μM) for 24 h. Cell viability was then performed using the MTT assay. The survival rate of neuroblastoma was significantly decreased by Rk1 in a dose-dependent manner. The half-maximal inhibitory concentration (IC_50_) was 12 μM in SK-N-BE(2), 15 μM in SH-SY5Y, and 30 μM in SK-N-SH, respectively (Figure 1C). Among three neuroblastoma cell lines, SK-N-BE(2) cells were more sensitive to Rk1 than SK-N-SH and SH-SY5Y, so SK-N-BE(2) cells were selected for subsequent studies. However, lower concentrations of Rk1 (<15 μM) showed no anti-growth effects on the BJ, CCD-1079SK, and HUVEC cells, as models of normal cells (Figure 1C). Additionally, the IC_50_ values of Rk1 in all neuroblastoma cell lines were relatively much lower than normal cells. Cell morphology imaging confirmed high apoptotic rates of three neuroblastoma cell lines in a dose-dependent manner (Figure 1D). Thus, these results indicate that Rk1 has a cytotoxic effect on neuroblastoma cells. 

### 2.2. Rk1 Triggers Apoptosis Causing Cell Death in SK-N-BE(2) Cells

**Figure 2:**
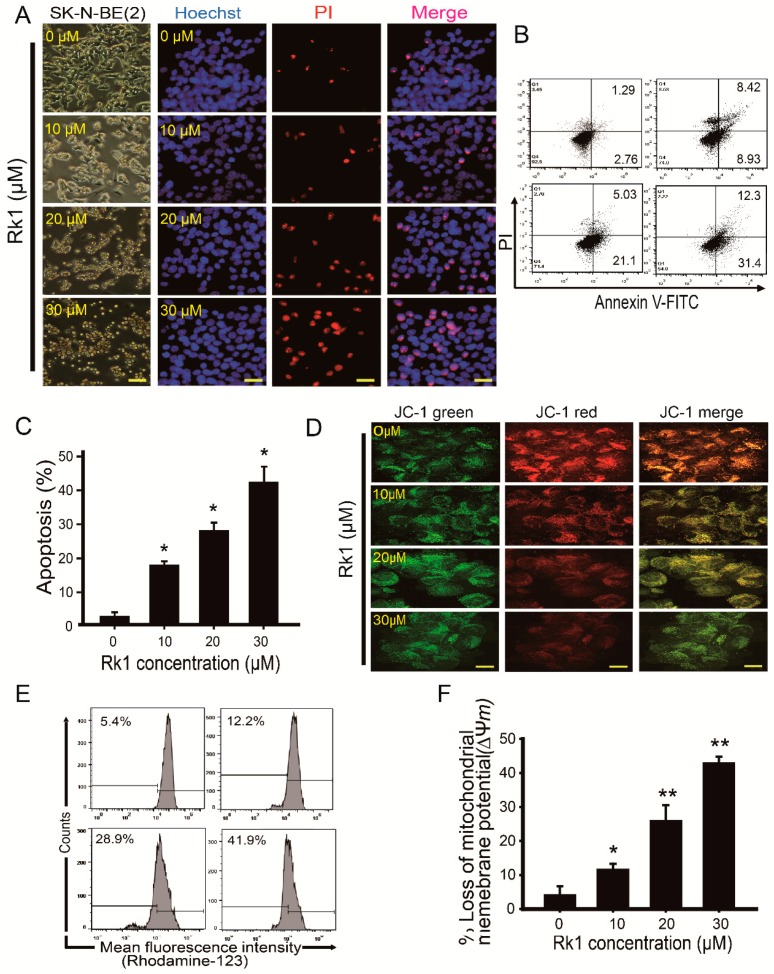
Rk1 induces apoptosis associated with mitochondrial membrane potential (∆ψm) loss in SK-N-BE(2) cells. (**A**) SK-N-BE(2) cells were exposed to Rk1 at various concentrations for 24 h and morphological changes were examined under a microscope. To assess apoptosis, Hoechst 33342/PI staining and flow cytometry analysis with FITC-conjugate Annexin V and PI (**B**) were used. The graph indicates the apoptosis level (%) in SK-N-BE(2) cells (**C**). Changes in mitochondrial membrane potential were analyzed using the JC-1 staining method (**D**) and rhodamine 123 dye (**E**) following Rk1 exposure at the indicated concentrations for 24 h. (**C**,**F**) Data are represented as mean ± SD three independent experiments. * *p* < 0.05 and ** *p* < 0.01 versus control. FITC, fluorescein isothiocyanate; PI, propidium iodide. Scale bar: 50 μm.

To investigate whether Rk1-induced decrease in cell viability is associated with apoptosis, SK-N-BE(2) cells were used because of its most strong effect for Rk1 treatment (Figure 1C). First, the morphological changes of SK-N-BE(2) cells were examined under a phase contrast light microscope with Hoechst 33342/PI staining. When treated with Rk1, it caused morphological changes from polygonal shape to a small round one, increased the number of floating cells, and reduced cell attachment. These effects were concentration-dependent. In untreated groups, cell nuclei were stained with a weak homologous blue, whereas in Rk1-treated groups, it was condensed with bright chromatin and shown as nuclear fragmentation (Figure 2A). Therefore, treatment with Rk1 induced apoptotic nuclear morphological changes in SK-N-BE(2) cells in a concentration-dependent manner. 

Next, we analysed the apoptosis rate of Rk1-treated SK-N-BE(2) cells by flow cytometry following Annexin V-FITC/PI double staining. Early and late apoptotic cell populations were shown as Annexin V^+^/PI^−^ and Annexin V^+^/PI^+^ cells, respectively, and both of them were considered apoptotic. Rk1 treatment with various concentrations (0, 10, 20, and 30 μM) significantly increased the percentage of apoptotic cells (4.05, 17.35, 26.13, and 43.7%, respectively) in a dose-dependent manner (Figure 2B,C). Taken together, these results indicate that Rk1 treatment causes apoptosis and induces cell death of SK-N-BE(2) cells.

### 2.3. Rk1 Reduces the Mitochondrial Membrane Potential (∆ψm)

To detect the change of Rk1-induced membrane potential in mitochondria, the sensitive fluorescent probes JC-1 and rhodamine 123 were used as indicators of mitochondrial dysfunction. Fluorescence was measured by fluorescence microscopy or flow cytometry (Figure 2D–F). As shown in Figure 2D, the control cells showed red fluorescence (JC-1 aggregates), indicating high ∆ψm. SK-N-BE (2) cells treated with 10 μM, 20 μM or 30 μM Rk1 for 24 h showed less red but increased green fluorescence, which means that membrane potential is low. Then, the cells were also treated with rhodamine 123, which is another marker for detection of mitochondrial membrane potential. Rhodamine 123 assay showed that Rk1 treatment induces mitochondrial membrane potential (∆ψm) loss (5.4, 12.2, 28.9, and 41.9% at 0, 10, 20, and 30 μM Rk1, respectively; Figure 2E,F). These results suggest that Rk1 induces apoptosis in SK-N-BE(2) cells by promoting mitochondrial dysfunction. 

### 2.4. Rk1 Induces G0/G1 Phase Arrest in SK-N-BE(2) Cells

**Figure 3:**
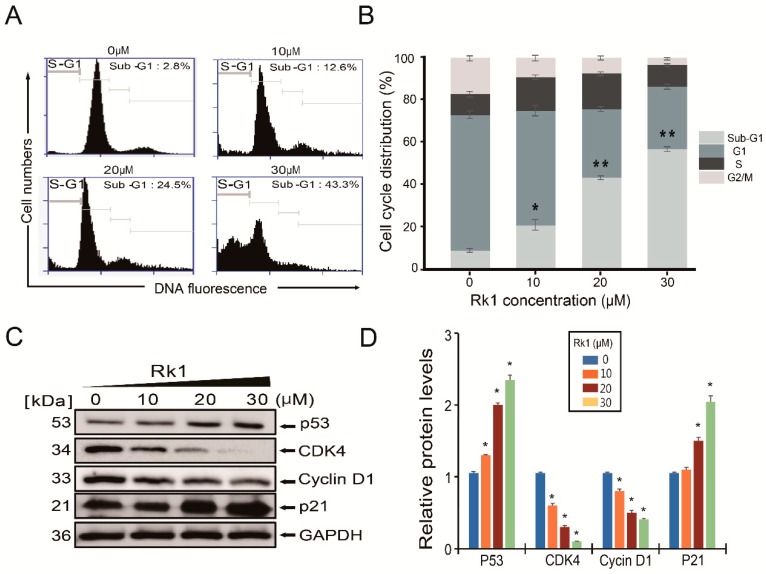
Rk1 blocks cell cycle progression of SK-N-BE(2) cells. (**A**,**B**) Cell cycle progression was analysed by flow cytometry following staining with PI. S-G1: sub-G1 phase. (**C**,**D**) The proliferation associated proteins, p21, Cyclin D1, p53 and CDK4 were analysed by western blotting. GAPDH was used as a protein loading control. Data are in (**B**,**D**) represented as mean ±SD three independent experiments. * *p* < 0.05 and ** *p* < 0.01 versus control. CDK4, cyclin dependent kinase-4.

To determine whether the cytotoxic effect of Rk1 on SK-N-BE(2) cells was caused by cell cycle arrest, cells were treated with various concentrations of Rk1 for 24 h, and the effect of Rk1 on cell cycle distribution was quantified by flow cytometry. As shown in Figure 3A,B, Rk1 markedly increased the sub-G1 hypodiploid cell population. Treatment of 10 μM, 20 μM, and 30 μM Rk1 induced increased accumulation of cells in the sub-G1 phase to 12.6%, 24.5%, and 43.3% compared to 2.8% in untreated control groups. The inhibitory effect on the growth of neuroblastoma cells with Rk1 treatment can be attributable by induction of G1 phase arrest. Because Cyclin D1, CDK4, p53 and p21 are key regulators of the transition from G0/G1 to S phase, the effect of Rk1 on the expression of these cell cycle regulators was investigated using western blot. 

As shown in Figure 3C,D, with Rk1 treatment, the protein levels of CDK4 as well as cyclin D1 were reduced significantly, whereas the levels of p21 and p53 were increased in SK-N-BE(2) cells. These results indicate that Rk1 blocks cell cycle progression at the sub-G1 phase, thereby inhibiting neuroblastoma cell proliferation. 

### 2.5. Rk1 Regulates Apoptosis-Related Protein and Caspase Activation in SK-N-BE(2) Cells

**Figure 4:**
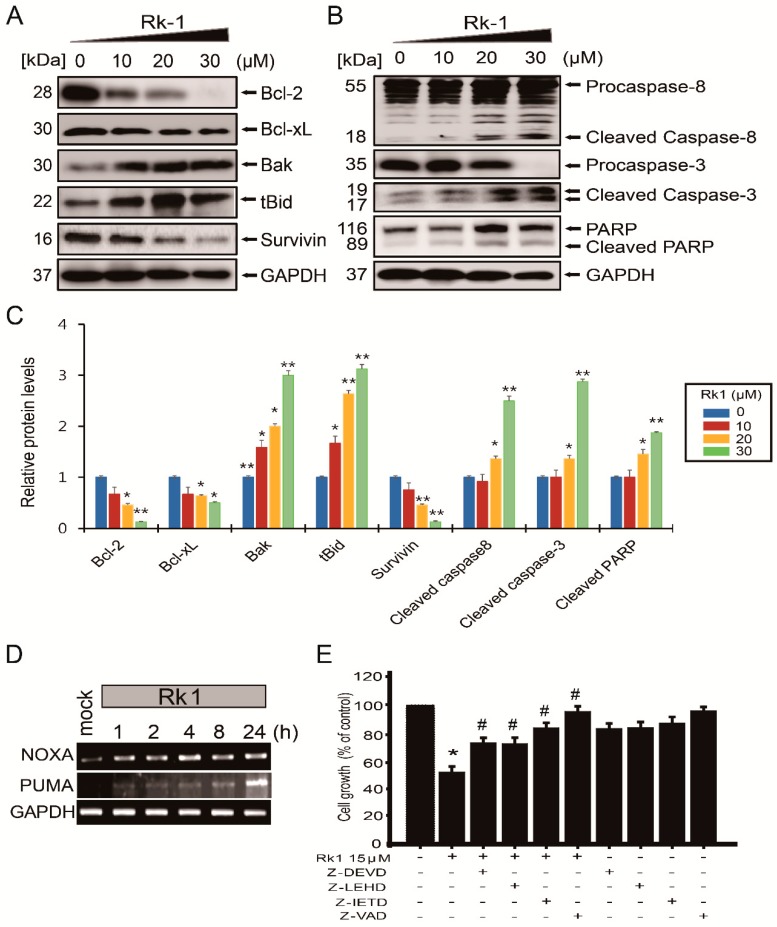
Rk1 induces apoptotic cell death in SK-N-BE(2) cells. (**A**–**C**) SK-N-BE(2) cells were treated with various concentrations of Rk1 for 24 h. Apoptotic markers were analysed by western blotting and GAPDH was used as a protein loading control. (**C**) Data are presented as mean ± SD three independent experiments. * *p* < 0.05 and ** *p* < 0.01 versus control. (**D**) The mRNA expression levels of the pro-apoptotic genes NOXA and PUMA were evaluated using RT-PCR and GAPDH was used as an internal control. (**E**) The effect of caspase inhibitors on apoptosis was determined by MTT assay. Caspase inhibitors reversed cell death by Rk1. Data are presented as mean ± SD three independent experiments. * *p* < 0.05 vs. control group. # *p* < 0.05 vs. Rk1 only treated group.

The activation of the caspase pathway and Bcl-2 family proteins has been reported to be the best known for the execution phase of apoptosis. Bcl-2 family proteins consist of members that either promote or inhibit apoptosis called as pro-apoptotic and anti-apoptotic proteins, respectively. These proteins are responsible for the release of cytochrome c from the mitochondria, which play a pivotal role in inducing apoptosis, and this mitochondrial damage causes an intrinsic apoptosis [20]. Therefore, we investigated whether the mechanism of the Rk1-induced cell death employed caspase proteins and Bcl-2 family genes by examining their expression by RT-PCR and western blot. As a result, apoptosis promoting genes such as Bak, tBID, PUMA, NOXA and PARP were upregulated by Rk1 treatment in a concentration-dependent manner (Figure 4A–D). In contrast, Rk1 dose-dependently inhibited the expression of genes inhibiting apoptosis such as Bcl-2, Bcl-xL and survivin (Figure 4A,C). We then investigated whether Rk1 induces caspase-dependent cell death in neuroblastoma cells by western blot and treatment with caspase inhibitors Z-DEVD (caspase-3 inhibitor), Z-IETD (caspase-8 inhibitor), Z-LEHD (caspase-9 inhibitor), and Z-VAD (pan-caspase inhibitor) for 3 h prior to 15 μM Rk1 exposure (Figure 4E). Apoptosis is mediated through initiator caspases (caspase-8 and -9), which leads to effector caspase (caspase-3) activation. In that respect, we found increased levels of cleaved caspase-3 and -8 with Rk1 treatment by western blot analysis (Figure 4B,C). Moreover, Rk1-induced apoptotic cell death was rescued by caspase inhibitors in SK-N-BE(2) cells (Figure 4E). Overall, these results suggest that Rk1 has an apoptotic effect by increasing the level of Bcl-2 family expression and activating caspases activities in SK-N-BE(2) cells.

### 2.6. Rk1 Induces Inhibition of Invasion but also Migration in Neuroblastoma Cells

**Figure 5:**
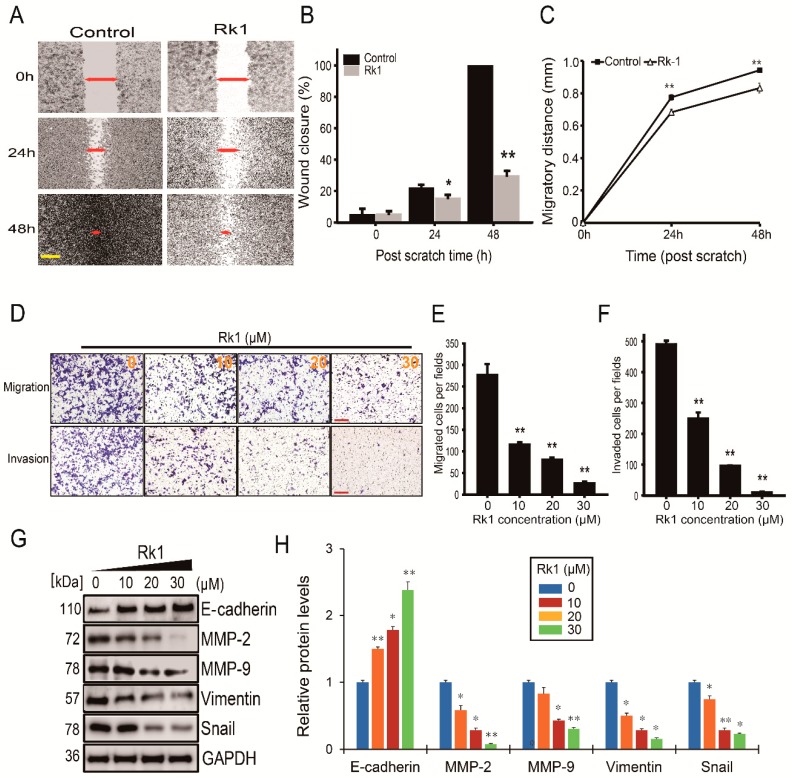
Rk1 inhibits EMT in SK-N-BE(2) cells. (**A**) Cell mobility was investigated by wound healing assay. Representative images from in vitro wound healing assays demonstrated that cell migration into the cell-free region (outlined) was significantly suppressed in the presence of Rk1, as compared to the control. Magnification of the images: 200×. Scale bar: 200 μm. (**B**,**C**) Bar graph illustrates percent wound closure (**B**) and migratory distance (**C**) was measured at 0 h, 24 h, and 48 h after cells were scratched. (**D**–**F**) Migration and invasion ability was analysed by transwell assay with non-coated membrane (upper panel) or with Matrigel-coated membrane (lower panel). (**D**) Magnification of the images: 100×. Scale bar: 100 μm. The migration (**E**) and invasion (**F**) of SK-N-BE(2) cells were significantly decreased in Rk1-treated groups. (**G**,**H**) The level of EMT-related proteins in SK-N-BE(2) cells was examined by western blot. Bar graphs in (**B**), (**C**), (**E**), (**F**), and (**H**) represented as mean ± SD three independent experiments * *p* < 0.05 and ** *p* < 0.01 vs. control group. MMP-2, matrix metalloproteinase-2; MMP-9, matrix metalloproteinase-9; E-cadherin, epithelial cadherin; GAPDH, Glyceraldehyde 3-phosphate dehydrogenase.

In cancer metastasis, cellular motility is regarded as an important factor. For that reason, the effect of Rk1 on migratory abilities of neuroblastoma cells was investigated by using wound healing and transwell migration assays. Rk1 treatment (12 μM) significantly decreased migration compared to the control groups (Figure 5A–C), as well as the number of migrated cells compared to the control groups dose dependently (Figure 5D upper panel and Figure 5E) in wound healing assays and transwell migration assays, respectively. Then, the effect of Rk1 on the invasion abilities was examined by Matrigel-coated Boyden Chamber and cell invasiveness was significantly decreased by Rk1 treatment 48 h after incubation (Figure 5D lower panel and Figure 5F). Overall, these results indicate that Rk1 treatment significantly decreases the migratory ability of neuroblastoma SK-N-BE(2) cells.

### 2.7. Rk1 Inhibits Tumor Progression by Blocking the EMT (Epithelial-Mesenchymal Transition) Process

Acquisition of the migratory characteristics of a mesenchymal-like state is believed to enhance the invasive capabilities of cancer cells. In the EMT process of tumor cells, expression of proteins promoting cell-cell contact such as E-cadherin is decreased, while expression of mesenchymal markers such as vimentin, MMP-2 and -9 is increased, resulting in cell migration and invasion ability is enhanced [21]. We, therefore, examined the expression of EMT-related genes in response to treatment with Rk1. E-cadherin levels were increased, whereas levels of vimentin, MMP-2, and MMP-9 were decreased. Moreover, the EMT-related transcription factor, Snail, also had reduced expression (Figure 5G,H). Collectively, these findings suggest that treatment with Rk1 possibly inhibits neuroblastoma cell migration and invasion through suppression of EMT.

### 2.8. Rk1 Inhibits Tumor Growth in a Xenograft Nude Mouse Model

**Figure 6:**
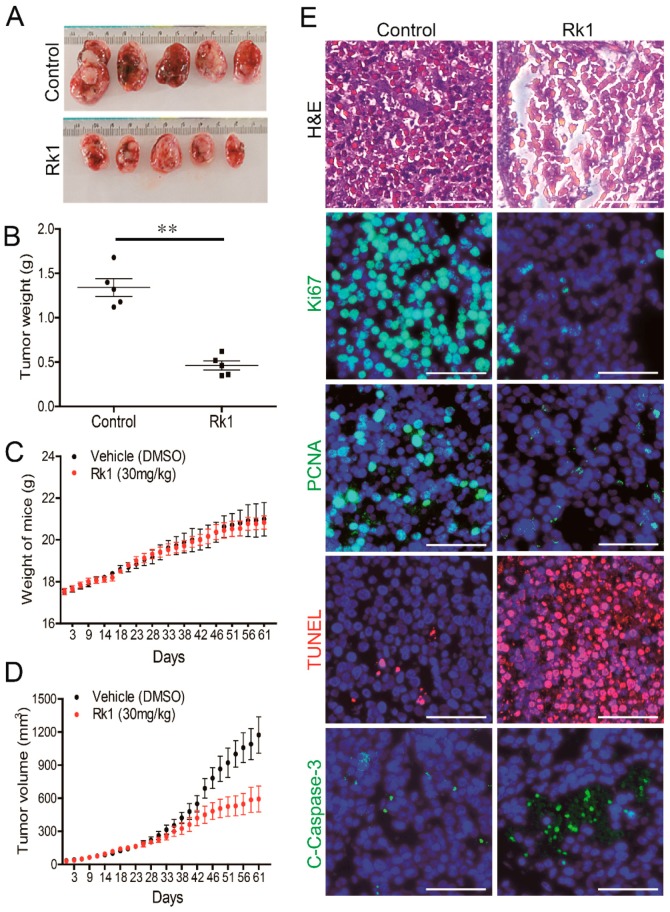
Rk1 reduces neuroblastoma growth in a xenograft mouse model. (**A**) Viable SK-N-BE(2) cells (3 × 10^7^) were subcutaneously injected into the flanks of nu/nu mice. After a week, mice were administered either vehicle (DMSO; n = 5) or Rk1 (30 mg/kg; n = 5) via intraperitoneal injection three times a week. Mice were sacrificed and tumors were collected 61 days after cell injection (**B**–**D**) tumor weight (**B**), mouse weight (**C**) and tumor growth (**D**) curve following Rk1 treatment (30 mg/kg) or vehicle control in SK-N-BE(2) xenografts. The statistical analysis of body weight, tumor weight and tumor volume are shown (* *p*< 0.05 and * *p* <0.01). (**E**) H&E staining in subcutaneous tumor samples. Ki-67, PCNA, TUNEL and C-caspase-3 expression were detected by immunohistochmical staining in xenograft tumor tissues. Magnification of the images: 200×. Scale bar: 50 µm.

To confirm the anti-cancer effect of Rk1 in vivo, we used the human neuroblastoma SK-N-BE(2) cells in an athymic nude mouse model. Interestingly, Rk1 treatment (30 mg/kg) significantly inhibited the growth of tumor (Figure 6A). The tumor weight (Figure 6B) and tumor volume (Figure 6D) were significantly decreased, but the mouse body weight (Figure 6C) was not changed, when compared to the control over 40 days. To further investigate whether Rk1 has anti-apoptotic effects on SK-N-BE(2) cells in vivo, the xenograft tumor tissues were fixed for confirmation by H&E staining and immunohistochemistry analysis of Ki-67, PCNA, cleaved caspase-3 and TUNEL. 

In Figure 6E, H&E staining shows the irregular structure in Rk1-treated tumor tissue. Furthermore, Rk1 treatment reduced the proliferation markers such as Ki-67 and PCNA but also increased the apoptotic markers such as TUNEL and cleaved caspase-3 in the Rk1-treated tumor tissues (Figure 6E). These data indicate that Rk1 significantly inhibits the growth of neuroblastoma in vivo by promoting cell death, likely by increased induction of apoptosis as well as reduced proliferation.

## 3. Discussion

Neuroblastoma is the second most common type of paediatric solid cancer, following brain tumors. Conventional chemotherapeutic agents often have cytotoxic effects in normal cells as well as cancer cells, and have limitations in treatment. To overcome the limitations of chemotherapy, additional novel approaches are required that increase survival and minimise serious side effects. The identification of anti-cancer substances from natural products can provide alternatives with higher safety and efficacy [22]. 

Several types of ginsenosides have been used for prevention and therapy in various cancers. However, there are not many studies conducted on RK1 effect, and its effects in neuroblastoma have not been reported. Therefore, in this study, we investigated the effects of Rk1 on apoptosis and metastasis in SK-N-BE(2) cells to determine whether Rk1 has potential as a novel anti-cancer drug for the treatment of neuroblastoma. 

Unlike normal cells, cancer cells undergo abnormal cell proliferation and strong growth, resulting in resistance to apoptosis [9,23]. For this reason, reducing cancer cell growth inhibiting ability can be an important method for suppressing cancer progression. Therefore, inhibition of tumor cell proliferation in tumor treatment is very important. We found that Rk1 have potent inhibitory effects on three neuroblastoma cell lines (Figure 1C,D, and Figure 2) and cell morphology observation (Figure 1D). The anti-proliferative effect of Rk1 on SK-N-BE(2) cells was mediated by G0/G1 phase arrest, which is due to inhibition of cyclin D1 and CDK4 expression and p21 and p53 activation (Figure 3), and induction of cell death through mitochondrial membrane potential (∆ψm) loss (Figure 2). Most anti-cancer agents induce cell death through two types of apoptotic pathways: the mitochondrial-mediated intrinsic and the extrinsic receptor-mediated pathway [23,24]. We found that Rk1 induces expression of caspase-3 and -8 as well as PARP but also it downregulates Bcl-2 and Bcl-xL and upregulates Bak and tBID (Figure 4A,C). This caspase-mediated apoptosis inducing ability of Rk1 has been reported in hepatocellular carcinoma HepG2 cells [18] and melanoma SK-MEL-2 cells [19]. Consistent with previous studies, our study in SK-N-BE(2) cells demonstrates that Rk1 activates both the intrinsic and extrinsic pathways.

Another important anti-apoptosis protein, survivin, is a novel member of the anti-apoptosis protein family [25,26] and is overexpressed in many human cancers, including gastric, colorectal, and bladder cancer [27,28]. Its overexpression has been reported to be a progressive disease and not good prognosis [29]. It has also been reported that survivin overexpression is associated with high-risk tumors and poor prognosis in neuroblastoma [13,14]. Therefore, inhibition of the expression of survivin may also be a good therapeutic strategy for the treatment of neuroblastoma. Our results showed that Rk1 inhibited the expression of survivin protein (Figure 4A,C). 

Metastasis is the phenomenon wherein cancer cells spread from the primary site to other parts of the body. Conversion from primary neuroblastoma to metastatic neuroblastoma is a complex multistage process involving cell attachment, migration, angiogenesis, immune escape and return to the target organ [22]. Clinical studies have shown that more than half of the patients with neuroblastoma have metastasized to the intracranial orbital sites, liver, cytoskeleton, bone marrow and lymph nodes [30]. The enhanced migration and invasion capabilities of neuroblastoma cells are critical features for the metastatic transformation process [31]. Hence, identification of the key molecules and pathways that control migration and invasion in neuroblastoma are important to understand neuroblastoma metastasis.

Tumor metastasis and infiltration occur in a multistep process involving the degradation of the extracellular matrix (ECM), causing cancer cells to spread to other organs. As a protein involved in this process, zinc-dependent proteinases such as MMP-2 and MMP-9 are known [32]. Numerous studies have implicated that MMP-9 and MMP-2 are involved in invasive, metastatic, and poor prognosis of various cancers [33,34,35,36], including neuroblastoma [37]. Therefore, inhibition of MMP-2 and MMP-9 has great potential in the treatment of neuroblastoma. Our results also showed that Rk1 significantly decreased MMP-9 and MMP-2 expression. In addition, Rk1 inhibited EMT by upregulating E-cadherin and downregulation of vimentin and Snail (Figure 5G,H). However, in order to observe more precisely the Rk1 effect on migration, a live cell tracking assay would also be needed.

Most of the anti-cancer properties assigned to Rk1 have been demonstrated in in vitro studies. Although some studies on the anti-oxidant, anti-inflammatory [38], anti-microbial, memory improvement [39], and neuroprotective [40] effects of Rk1 in animals are reported, the effects of Rk1 on neuroblastoma in vivo have not been previously studied. Our xenograft experiments demonstrating that the development of tumors in animals was markedly suppressed by Rk1 treatment (30 mg/kg) provide strong evidence supporting the anti-cancer potential of Rk1 (Figure 6). More importantly, to our knowledge, this is the first time the potential mechanisms underlying these anti-cancer effects of Rk1 have been identified. 

In conclusion, our results clarified that Rk1 suppressed the cell viability and induced Go/G1 phase arrest and cellular apoptosis. Moreover, intrinsic and extrinsic cell death pathways were involved in Rk1-induced cell death in neuroblastoma cells. These results suggest that Rk1 can be a safe cancer therapeutic drug because it induces apoptosis in cancer cells and reduces migration/invasion ability through reduction of EMT signaling, even at low concentrations. However, studies on the efficacy and safety for humans are required to investigate the therapeutic potential of Rk1 for cancer.

## 4. Materials and Methods

### 4.1. Ginsenoside Rk1 Isolation

The Rk1 (purity > 98%) was prepared by a transformation of PPD-type ginsenosides (Rb1, Rb2, Rc, and Rd: PPD mix) extracted from Korean ginseng using acid-heat treatment [41]. PPD mix was converted to Rg3-mix [20(*S*)-Rg3, 20(*R*)-Rg3, Rk1, and Rg5] through a stepwise hydrolysis of the outer and inner glucose or arabinose of C-20, [(Rb1, Rb2, Rc, Rd) → Rg3-mix]. One gram of crude Rg3-mix was purified using a recycling preparative HPLC system (LC-5060, Japan Analytical Industry Co., Ltd., Tokyo, Japan) with a UV/refractive index detector as well as a reverse-phase column (Octadecylsilane) (500 by 20 mm; inside diameter, 15 μm). An isocratic solvent system of CH_3_CN and deionised H_2_O (6:4) was used, and the detection wavelength was set at 203 nm. Finally, 65 mg of 20(*S*)-Rg3, 23 mg of 20(*R*)-Rg3, 31 mg of Rk1, and 37 mg of Rg5 were purified (Figure 1B). For experiments, DMSO was used to dissolve Rk1 and it was diluted in medium for cell culture as needed. The control group received the same volume of the vehicle solution without Rk1 treatment.

### 4.2. Reagents and Antibodies 

Fetal bovine serum (FBS), antibiotics (penicillin-streptomycin), and Dulbecco’s modified Eagle’s medium (DMEM) were obtained from Gibco-BRL (Grand Island, NY, USA); 3-(4, 5-dimethylthiazol-2-yl)-2; dimethyl sulfoxide (DMSO) and in situ Cell Death Detection Kit (TMR red) from Sigma-Aldrich (St. Louis, MO, USA); 5-diphenyltetrazolium bromide (MTT) from Molecular Probes (Eugene, OR, USA). The following antibodies were used: GAPDH, Bcl-2, survivin, caspase-3, caspase-8, cleaved caspase-3, PCNA and p53 (Cell Signalling, Danvers, MA, USA), PARP, and Bcl-xL (Santa Cruz Biotechnology, Dallas, TX, USA), matrix metalloproteinase (MMP)-2 (Bioss Antibodies Inc., Woburn, Massachusetts, USA), MMP-9, Ki-67, and alexa 488/594 (Abcam, Cambridge, UK), and p21, cyclin D1, CDK4, Bak, tBID, E-cadherin, vimentin, and snail (R&D Systems, Minneapolis, MN, USA). Annexin V-FITC Apoptosis Detection Kit, propidium iodide (PI), Hoechst 33342 staining kit, Matrigel, and various caspase inhibitors were purchased from BD Biosciences (San Jose, CA, USA). 

### 4.3. Cells and Culture Conditions

SK-N-SH was purchased from the Korean Cell Line Bank (Seoul, Korea) and other cell lines (SK-N-BE(2), SH-SY5Y, BJ, CCD-1079SK, HUVEC) were purchased from the American Type Culture Collection (ATCC, Manassas, VA, USA). Cells were cultured in DMEM supplemented with 10% FBS and penicillin/streptomycin at 37 °C and 5% CO_2_.

### 4.4. Cell Viability Assay 

Cell viability was assessed by the MTT method. Briefly, all cells (1 × 10^4^ cells/well) were seeded in 96-well plates (SPL Life Science, Phocheon-si, Korea), then exposed to various concentrations of Rk1 for 24 h. The medium was then removed and the cells were incubated for 3 h at 37 °C with fresh medium containing 0.5 mg/mL MTT. The medium was then removed, and cells were incubated with fresh medium containing 0.5 mg/mL MTT for a further 3 h at 37 °C. The formazan products in cells were dissolved in 200 μL of DMSO and measured spectrophotometrically at 570 nm using a microplate reader Synergy^TM^ 2 (BioTek Instruments Inc., Winooski, Vermont, USA). All experiments were performed in triplicate. Cell viability is expressed as a percentage of the control. 

### 4.5. Cell Cycle Analysis 

SK-N-BE (2) cells were seeded onto 6-well plates at a density of 5 × 10^5^ cells/well and treated with various concentrations of Rk1 for 24 h. Floating and adherent cells were collected with trypsin-EDTA (Sigma-Aldrich, St. Louis, MO, USA) and fixed in 70% ethanol overnight at −20 °C. The cells were washed with cold PBS and stained with 10 μg/mL of PI and incubated at 37 °C for 3 h. Then, 10,000 fluorescent events were measured and analysed with an Accuri C6 flow cytometer (BD Biosciences, San Jose, CA, USA).

### 4.6. Annexin V/PI Staining 

Apoptotic cell death was determined using an annexin V-fluorescein isothiocyanate (FITC)/PI apoptosis detection kit. Briefly, 5 × 10^5^ cells were treated with Rk1 (0, 10, 20, and 30 μM). After 24 h, the cells were harvested, washed with PBS, and stained with 5 μL of Annexin V-FITC and PI (1 mg/mL). The stained cells were analysed by flow cytometry (BD Biosciences, San Jose, CA, USA). The number of apoptotic cells was counted and presented as a percentage of the total cell count. 

### 4.7. Mitochondrial Membrane Potential (∆ψm) Assay 

∆ψm level was measured with rhodamine 123 and JC-1 dyes (Enzo Life Sciences, Inc., Farmingdale, NY, USA). Briefly, 1 × 10^5^ cells in 12-well culture plates (SPL) were incubated with Rk1. After 24 h, the supernatant was removed, and cells were stained with 0.1 μg/mL rhodamine 123 or 5 μg/mL of JC-1 for 30 min at 37 °C. The cell pellet was suspended in PBS and imaged. The intensity of rhodamine 123 staining was determined by Accuri C6 flow cytometry. The lack of rhodamine 123 staining showed loss of mitochondrial membrane potential (∆ψm).

### 4.8. Reverse Transcriptase-Polymerase Chain Reaction (RT-PCR) 

Total RNA was extracted using TRIZOL reagent (Invitrogen, Carlsbad, CA, USA) and complementary DNA (cDNA) was synthesised using GoScript reverse transcription system (Promega, Madison, WI, USA). All samples were assayed in triplicate. The relative expression of each gene was normalised to that of *GAPDH* loading control using ImageJ program (NIH). The PCR products were electrophoresed on a 1.2% agarose gel and visualised on a UV transilluminator using Red Safe (iNtRON Biotechnology, Seongnam, Gyeonggi, South Korea). The following primer sequences were used: (1) human *NOXA* (forward) 5′- CGGAGATGCCTGGGAAGAA-3′C and (reverse) 5′-AGGTTCCTGAGCAGAAGAGT-3′; (2) human *PUMA* (forward) 5′- AGTGTCCTGCGGCCTCTG-3′ and (reverse) 5′- GGAGTCCCATGATGAGATTGT -3′; and (3) human *GAPDH* (forward) 5′-GAGTCAACGGATTTGGTCGT-3′ and (reverse) 5′-GACAAGCTTCCCGTTCTCAG -3′.

### 4.9. Western Blot Analysis 

Whole cell protein lysates were prepared, and the lysates were analysed using western blot assays as previously described [42]. In brief, cell lysates were separated on SDS/12% PAGE gels and transferred onto PVDF membranes (Millipore). The membranes were then probed with the primary antibodies overnight, following which they were incubated with horseradish peroxidase (HRP)-conjugated secondary antibodies. Blots were developed using an ECL solution kit (GE Healthcare, Piscataway, NJ, USA).

### 4.10. Wound-Healing Assay 

Neuroblastoma cells were seeded in 12-well plates. After 24 h, the monolayer was scratched with a sterile 200 µL pipette tip. Cells were washed with PBS and then exposed to Rk1 in DMEM without FBS. After drug treatment for 24 h and 48 h, the wounded area was observed and imaged using a light microscope (Leica Microsystems GmbH, Wetzlar, Germany). The experiment was repeated three times. 

### 4.11. Cell Migration and Cell Invasion Assay 

The migration and invasion assays were performed using transwell chamber inserts with a pore size of 8.0 μm (SPL). For the migration assay, 5 × 10^4^ cells and various concentrations of Rk1 were added to the upper chamber with the non-coated membrane. For invasion assays, the upper chamber with the Matrigel coated membrane was diluted with serum free medium. In both tests, cells were suspended in 200 μL serum free medium and seeded in the upper chamber. In the lower chamber, 800 μL of complete medium was added. After 48 h, the cells were fixed with cold methanol and stained with 0.2% crystal violet. Invading cells were analysed using a light microscope (Leica Microsystems GmbH, Wetzlar, Germany). Cells in five random fields were counted and expressed as the average number of cells/fields. 

### 4.12. Tumour Xenograft Studies 

4–5 weeks old Male BALB/c homozygous nude mice (~17 g body weight) were purchased from NARA Biotech (Seoul, Korea). All experimental procedures were approved by the Chonbuk National University Institutional Animal Care and Use Committee. (Permit no. CBNU 2018-013). The methods have been performed in accordance with the approved guidelines and regulations. The mice were maintained in a specific pathogen-free environment. SK-N-BE(2) cells (3 × 10^7^) in 100 μL (PBS: Matrigel = 1:1) were injected subcutaneously into the right flank. One week later, tumor injected mice were randomly divided into Rk1 and control groups (n = 5 per group). The control or Rk1 group was injected with DMSO or 30 mg/kg Rk1 intraperitoneally thrice a week, respectively. Body weights and tumor volumes were measured under anesthesia with isoflurane during Rk1 treatment. The tumor volumes were calculated as {V= π/6 × (length × width × height)} as previously described [42]. Mice were sacrificed 60 days after tumor inoculation.

### 4.13. Histology 

For immunohistochemistry, paraffin-embedded samples were sliced into 4 µm sections, deparaffinized and underwent an antigen retrieval procedure by boiling in 10 mM Citric acid (pH 6.0) for 15 min using a pressure cooker. Sections were blocked with PBST with 5% normal goat serum for 30 min and incubated with anti-Ki-67 (1:500), anti-PCNA (1:500), anti-Cleaved Caspase-3 (1:250) antibodies overnight at 4 °C. After PBS washes, sections were incubated with Alexa Fluor^®^ 488 or 594 at RT for 1 h. For TUNEL staining, sections were incubated with the reaction mixture that contains TdT and TMR-dUTP. For immunofluorescence, the density of 5 × 10^5^ cells/well SK-N-BE(2) cells were seeded onto 25-mm circular coverslips (SPL) and treated with Rk1 for 24 h. The cells were rinsed with PBS twice, followed by incubation with 1 μg/mL Hoechst 33342/PI reagent at 37 °C for 30 min. The cells were then fixed with cold methanol for 10 min at RT and washed with PBS. The stained cells were photographed using a fluorescence microscope (CELENA S, Logos Biosystems, Anyang-si, Korea). 

### 4.14. Statistical Analysis 

All data were presented as mean ± SD of at least three independent experiments, each performed at least in triplicate. All statistical analyses were determined using SPSS 12.0 software (Chicago, IL, USA). The differences between two independent groups were analyzed by two-tailed Student’s *t*-test. All comparisons were considered statistically significant at *p* < 0.05(*) and *p* < 0.01(**).
